# A comparative study of mechanical resistance of two reciprocating files

**DOI:** 10.4317/jced.55487

**Published:** 2019-03-01

**Authors:** Dario Di Nardo, Massimo Galli, Antonio Morese, Marco Seracchiani, Valerio Ferri, Gabriele Miccoli, Gianluca Gambarini, Luca Testarelli

**Affiliations:** 1 DDS, PhD, Department of Oral and Maxillo-Facial Sciences, Sapienza University of Rome, Italy; 2 MD. Department of Oral and Maxillo-Facial Sciences, Sapienza University of Rome, Italy; 3 DDS Department of Oral and Maxillo-Facial Sciences, Sapienza University of Rome, Italy; 4 DDS, PhD student. Department of Oral and Maxillo-Facial Sciences, Sapienza University of Rome, Italy

## Abstract

**Background:**

To evaluate the cyclic fatigue resistance of two different Nickel-Titanium instruments, Reziflow (Komet, Brasseler GmbH & Co., Lemgo, Germany) and WaveOne Gold (Dentsply Maillefer, Ballaigues, Switzerland).

**Material and Methods:**

Two groups of 20 different NiTi endodontic instruments of identical tip size of 0.25 mm were tested; Reziflow and Wave-One Gold primary. Cyclic fatigue testing was performed in a stainless steel simulated root canal manufactured by reproducing the instrument’s size and taper. A simulated root canal with a 90 degrees angle of curvature and 5 mm radius of curvature was constructed for the instruments tested. The centre of the curvature was 5 mm from the tip of the instrument and the curved segment of the canal was approximately 5 mm in length. Both the instruments were used in the same preset program specific for the WaveOne instruments. Each instrument was rotated until fracture occurred and the time to fracture (TtF) and the length of the fractured fragment were recorded. Means and standard deviations of TtF and fragment length were calculated and data were subjected to statical analysis (*P*<0.05).

**Results:**

Statistically significant differences (*P*<0.05) were noted between Reziflow and WaveOne Gold instruments. There were no significant differences (*P*>0.05) in the mean length of the fractured fragments between the instruments.

**Conclusions:**

Rezifllow instruments were associated with a significantly higher cyclic fatigue resistance than WaveOne Gold instruments.

** Key words:**Endodontic instruments, NiTi alloy, Reciprocating motion, Cyclic Fatigue, Heat treatment.

## Introduction

Since the early 1990, the introduction of nickel-titanium (NiTi) alloy for the endodontic treatment has improved the shaping procedures in the root canal preparation, making it easier and faster (1-3). Unfortunately, despite the superior mechanical properties of the NiTi alloy, the risk of intracanal separation of the instruments increased due to the continuous rotation and higher speed used (4-11).

The multifactorial origin of the NiTi instruments separation has been demonstrated in various clinical and experimental studies. According to these studies, cyclic fatigue is one of the most relevant cause of intracanal instruments breakage (12-14). Starting from the manufacturing process, each instrument shows some irregularities and defects and their distribution influences the fracture resistance of the resulting product (15). Indeed the fatigue failure begins with the formation of surface microcracks which seems to arise from the described irregularities. The microcracks deepening, as a result of each loading cycle, leads to the complete instrument separation (16-18).

In the last years, clinicians and manufacturers tried to find different ways to reduce the risk of intracranial separation, making the instruments more resistant to both flexural and torsional stress. To reach this aim, the development has followed mostly three different ways: the changing in the instruments design, the heat treatments of the alloy and the use of different kind of motions (19-23). Reciprocating motions has grown in popularity in the last ten years and many researches were published in the last years (24,25). All studies clearly demonstrate that reciprocation motion has a significant influence on cyclic fatigue resistance, comparing the same instruments in reciprocating and continuous motion (26,27).

Reziflow (Komet, Brasseler GmbH & Co., Lemgo, Germany) are new traditional NiTi instruments, with an S-shaped cross sectional design. thought to be used in counter-clock wise (CCW) reciprocating motion.

Wave-One Gold (WOG) (Dentsply Maillefer, Ballaigues, Switzerland), instruments are heat treated NiTi alloy instruments with two different cross-section, a modified convex triangular cross-section at the tip and a convex triangular cross-section in the middle and coronal portion, born to be used in counter-clock wise (CCW) reciprocating motion.

To date there are no studies comparing the resistance of WOG and Reziflow instruments to cyclic fatigue in a stainless steel artificial canal. Therefore the aim of this study is to compare the resistance to cyclic fatigue of WOG and Reziflow instruments in an artificial canal with a 90° curvature and a radius of 5 millimeters. The null hypothesis was that there are no differences between the two tested instruments in cyclic fatigue lifespan.

## Material and Methods

A total of 40 new NiTi instruments 25 mm in length were tested in this study: 20 Wave One Gold Primary, tip size 25 and variable taper (Dentsply Maillefer, Ballaigues, Switzerland) and 20 Reziflow (Komet, Brasseler GmbH & Co., Lemgo, Germany) tip size 25 and taper 06. All instruments were of the same length and used in the same reciprocating motion, the only difference was in the three-dimensional design. All of them had been previously inspected for morphological defects or any visible signs of deformations using a stereomicroscope at x20 magnification. None of them were discarded. Both the instruments were used in the same preset program specific for the WaveOne Gold instruments, because Reziflow has no preset motion and manufacturer declare its adaptability to preexisting CCW reciprocating motion.

In the present study, it was used a cyclic fatigue testing device already featured in previous studies (28,29). The device consists of a main platform to which is connected the electric handpiece and a stainless-steel block containing the artificial canals. The electric handpiece was mounted on a mobile device to allow for precise and reproducible placement of each instrument inside the stainless steel canal, ensuring that each instrument reached the same depth (18 mm). The same artificial root canal with a 90 degrees angle of curvature and 5 mm radius of curvature was used for all the tested instruments. Moreover the whole procedure has been performed by the same operator, to keep as low as possible the variability during the testing procedure.

All instruments were inserted at the same length (18mm) and then rotated in reciprocating motion until fracture occurred. For each instrument, the time was stopped as soon as the fracture was detectable and registered with a 1/100 sec chronometer. Time to fracture for each instrument was recorded (TtF).

**Figure 1:**
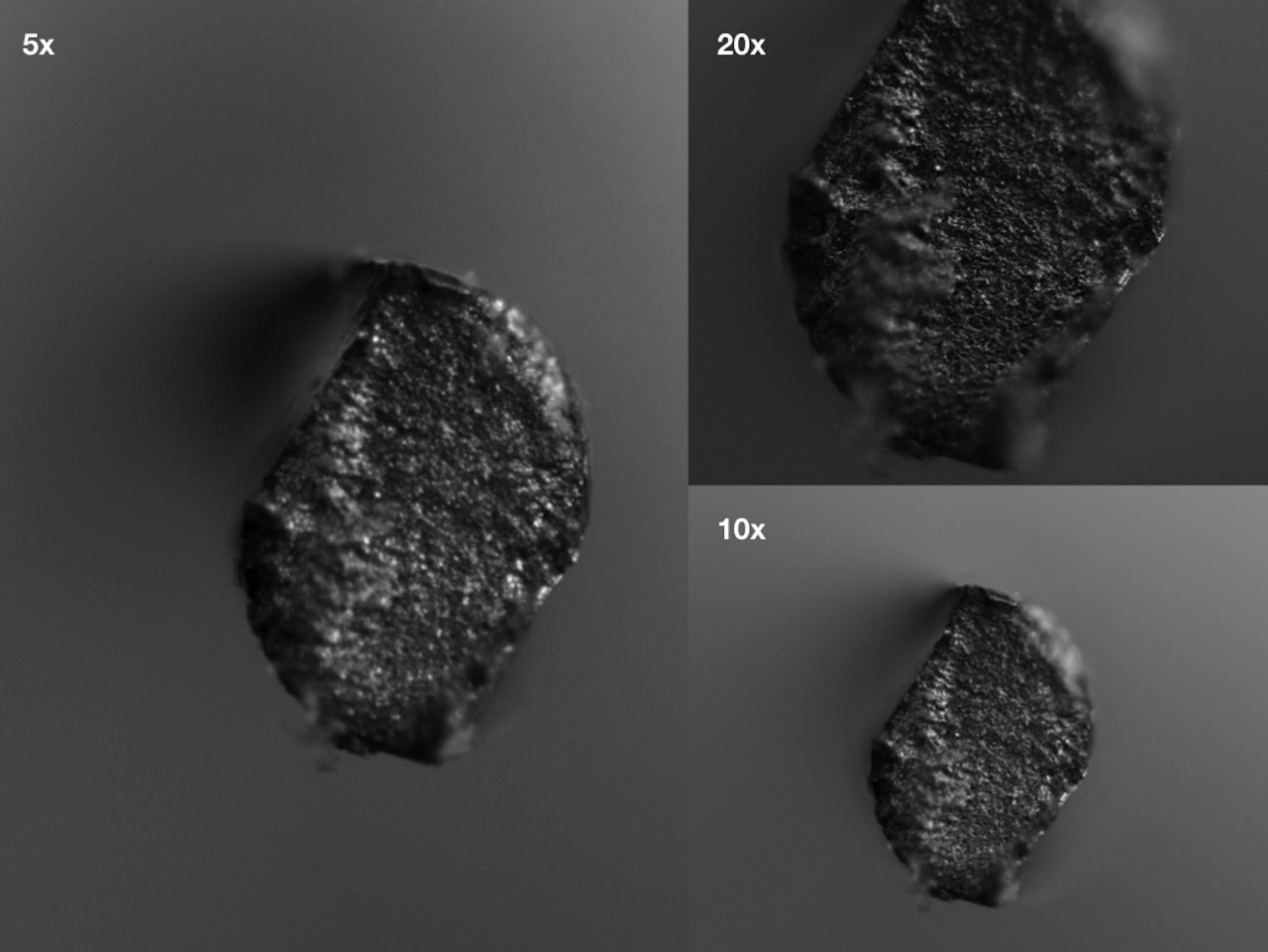
Reziflow’s fractured site at 5x, 10x and 20x magnification (Zeiss Scope A1, Oberkochen, Germany).

**Figure 2:**
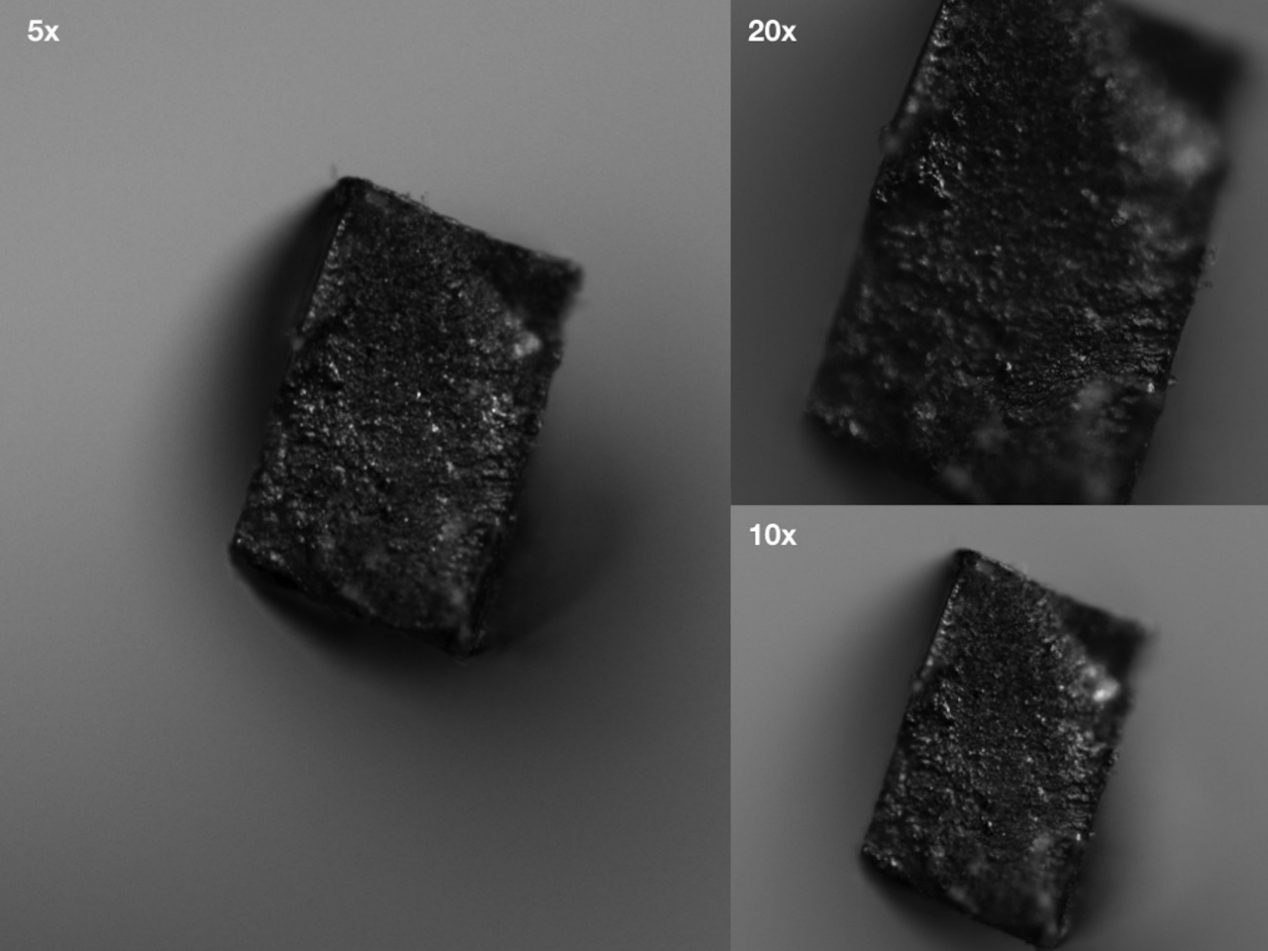
WaveOne Gold’s fractured site at 5x, 10x and 20x magnification (Zeiss Scope A1, Oberkochen, Germany).

Fragments were collected, measured by using a digital caliber and subject to optical microscopic analysis (Figs. 1,2).

The data were collected and mean and standard deviation were calculated. Differences among groups were statistically evaluated with an analysis of variance test ANOVA (significance level was set at *P* < 0.05). Data were statistically analyzed using the SPSS 17.0 software (SPSS Incorporated, Chicago, IL, USA).

## Results

Mean values for fragment length, showing no statistically relevant differences (*p* >0,05), demonstrate that there is no difference in the insertion of the instruments inside the stainless steel canal and therefore no differences in the portion of the instrument subjected to the stresses ( Table 1).

**Table 1:**
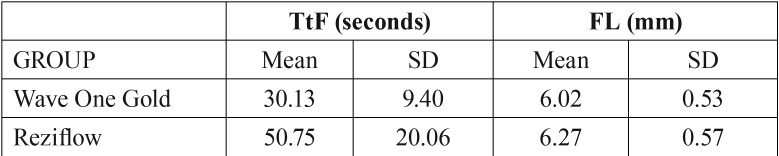
Time to fracture (seconds) and length (mm) of fractured fragments of instruments during cyclic fatigue testing.

Mean values for time to fracture for Reziflow instruments were 50,75 seconds (SD +/- 20,06) and for WOG instruments were 30,13 seconds (SD +/- 9,40). Statistical analysis found significant differences between the two instruments (*p* <0,05).

## Discussion

The two instruments selected for the study were both designed for use in CCW reciprocating motion. The instruments have the same tip size #25, but a different taper 0.08 for the coronal part, 3 mm from the tip, for the WOG and 0.06 constant taper for the Reziflow.

The test results showed significant differences: these results could have been influenced by three main different factors: design, alloy and motion. The recent literature demonstrated the importance of the angles of reciprocating motion in the cyclic fatigue life of NiTi rotary instruments (27). Although the Wave One motion is not clearly disclosed by the manufacturers, the present study was not influenced by the reciprocating angles, because both the instruments have been rotated using the Wave One proprietary motion. It must be explained that the reciprocating movement’s main aim is to reduce torsional loads and consequently torsional failure: the effect on flexural stresses is probably less evident, resulting in a significant improvement during the cyclic fatigue test (26,27).

The instruments were made from different alloys: thermal treated, XX-Wire, for Wave One Gold and traditional NiTi alloy for Reziflow. According to previous study, cyclic fatigue resistance can be influenced by the alloy and the manufacturing process of the instruments (30). Thus the results of this study can be influenced by their metallurgical composition and related behavior.

Another possible explanation of the test’s results is related to the different design and cross-section of the tested instruments. Reziflow has an S-shaped cross sectional design, similar to Reciproc and M-Two instruments. WaveOne Gold instruments have a modified convex triangular cross-section at the tip and a convex triangular cross-section in the middle and a coronal portion that is similar to the cross-section of the ProTaper instruments (31,32). Nevertheless the influence of instrument’s design on the cyclic fatigue resistance is controversial, and is still unclear how the cross-sectional design can influence the stress on the instruments. (33-36) Anyway most recent studies demonstrated that the resistance of rotary instruments is influenced by the quantity of metal mass: it is reported that the S-shape cross sectional design is characterized by a lower cross sectional metal mass that is related with an higher fatigue resistance (31). The influence of the design in the cyclic fatigue lifespan has been considered also in other studies (32).

The null hypothesis can be rejected, as the result of the test that showed statistically significant differences between the two instruments in cyclic fatigue lifespan. However, each of the different design and manufacturing features could be responsible for fatigue resistance, but it is not clear which of them is most correlated with instrument’s impairment, since the different features act simultaneously.

Wave One and Reziflow showed statistically significant differences in cyclic fatigue test. Since motion was the same, differences could be related to two factors: the different cross sectional and\or the different thermal treatment of the two instruments. However, further studies are needed to better evaluate the specific role of the different cross-sectional design and the thermal treatment of the alloy, in improving fatigue resistance to fracture.
